# Overlapping genes: a window on gene evolvability

**DOI:** 10.1186/1471-2164-15-721

**Published:** 2014-08-27

**Authors:** Maxime Huvet, Michael PH Stumpf

**Affiliations:** Theoretical Systems Biology Group, Department of life sciences, Imperial College London, London, SW7 2AZ UK

**Keywords:** Overlapping genes, Evolution, Expression regulation, Operon

## Abstract

**Background:**

The forces underlying genome architecture and organization are still only poorly understood in detail. Overlapping genes (genes partially or entirely overlapping) represent a genomic feature that is shared widely across biological organisms ranging from viruses to multi-cellular organisms. In bacteria, a third of the annotated genes are involved in an overlap. Despite the widespread nature of this arrangement, its evolutionary origins and biological ramifications have so far eluded explanation.

**Results:**

Here we present a comparative approach using information from 699 bacterial genomes that sheds light on the evolutionary dynamics of overlapping genes. We show that these structures exhibit high levels of plasticity.

**Conclusions:**

We propose a simple model allowing us to explain the observed properties of overlapping genes based on the importance of initiation and termination of transcriptional and translational processes. We believe that taking into account the processes leading to the expression of protein-coding genes hold the key to the understanding of overlapping genes structures.

## Background

The extent to which the protein coding regions of different genes overlap is a striking feature of many genomes. When two genes overlap the same portion of the DNA codes for the constituent amino acids of the two, typically different, proteins involved in the overlap. These overlapping structures can be observed in viruses [1], prokaryotes [2], and also eukaryotes [3]. Several previous studies have already made attempts at characterizing overlapping genes (OGs) in bacteria. These revealed a correlation between the number of genes in an organism and the number of OGs: approximately a third of genes are involved in an overlap arrangement [4]. Co-oriented overlapping gene pairs (OGPs) represent the vast majority of these observed structures, and very few incidences of OGPs with divergent orientation have been found [5]. In addition, the majority of OGPs overlap by 2 nucleotides, and more generally have a “+2” frame shift, and hardly any “+0” frame shift cases (overlap of three, or multiples of three, nucleotides) are observed [4, 6–8].

Different hypotheses have been put forward to explain the role and/or benefit of this configuration, including: (i) improved genome compaction [1, 9, 10], and (ii) implications for translation regulation through the mechanism of translational coupling [1, 10, 11]. The development of the genome compaction model is closely related to properties of those genomes in which the first overlaps were observed: viruses. In viruses a large proportion of genes overlap, which leads to a significant reduction in the number of nucleotides needed for encoding genes, and reduces the time and material needed to generate new genetic material for the creation of new viruses. The second hypothesis involving translational coupling is based on the molecular properties of the translation machinery. Usually protein-coding genes are transcribed into RNA, which is then (potentially after further editing) translated into a protein by the translation machinery. In bacteria some protein coding genes form sets (operons), which are transcribed together leading to polycistronic RNA. This is then translated into as many different proteins as there were genes in the operon. Diverse lines of evidence suggest that the production of these proteins may not be independent. After translating the sequence corresponding to one gene, the same translational machinery could continue and directly start the translation of the following gene [11, 12]. This process, referred to as translational coupling, is dependent on the distance between the successive protein coding sequences in the polycistronic mRNA. The presence of OGs could impact on the feasibility of this process. Despite the accumulation of information related to OGs, the possible biological implications are still far from being understood. However, this has not stopped researchers from developing mechanistic models that lead to the creation of these structures. The models of the underlying mechanism are based on modification of the genes’ boundaries. One model is in favor of STOP codon position change [13], while a more recent one makes the case for a START codon position change [14]. In both cases the models rely on the possibility for a gene to lose its START or STOP codon due to a mutation leading to the use of an alternative START or STOP codon.

Previously reported work mainly falls into two categories: (i) statistical analyses of large numbers of OGs considered together and often compared with the observed frequencies of START and STOP codons along the different reading frames surrounding the genes [4, 5, 13, 14]; and (ii) “anecdotal” analyses of specific OGPs, usually related to a specific species or biological system [6, 15–17], together with an analysis of the extent of the overlap through evolution. Only a few have looked at the phylogenetic profiles of large number of OGPs [8, 18]. Developing this type of approach has the potential to characterize the salient features of OGs, such as the stability of the overlap, and identify the scenarios under which the creation of an overlap is not deleterious.

In this paper we describe an analysis of the genomic organization of OGs in bacteria. By taking a comparative perspective (using data from almost 700 bacterial genomes) we are able to shed light on the evolutionary dynamics of this important and widely shared genomic structure. In contrast to earlier analyses [4, 9] we argue that OG structures have high plasticity, and (within the scope of suitable arrangements, which we will also discuss at some length) can easily be gained or lost. Finally, we discuss how failure to properly account for genomic structures such as operons in the analysis of overlapping genes can (and indeed has) lead to misleading interpretations.

## Results

### Properties of OGs in 699 species

**Figure 1:**
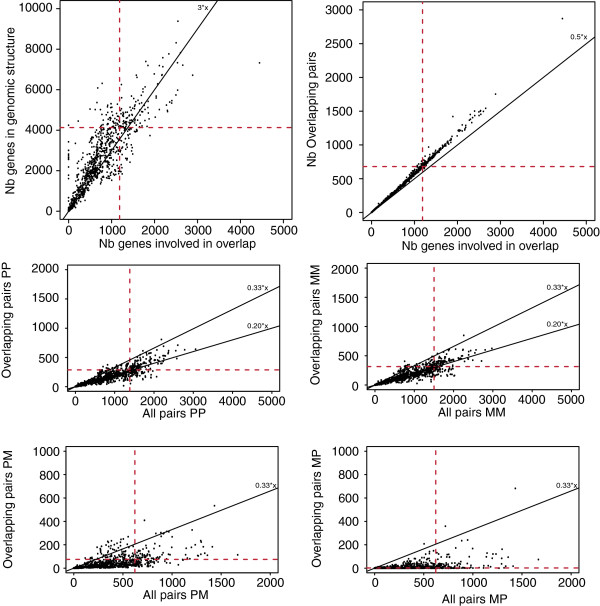
**Characteristics of overlapping gene pairs in 699 bacterial species.** The red dashed lines represent the values extracted from *E. coli*. Reference slope are drawn to facilitate the comparison of the different plots. On the second and third line are presented information related to overlapping genes with specific orientation. “P” stands for genes annotated on the plus strand and “M” on the minus strand.

In this paper we have analysed all pairs of OGs from 699 organisms. The genomes of interest encode a total of 2,272,519 protein-coding genes with 381,783 OG pairs (OGPs). Among chromosomes and plasmids, the numbers of OGPs range from 0 to almost 3000 (680 in *Escherichia coli K12 MG1655*). In total, just 53 of these structures have no OGPs and most of them contain less than 10 genes (103 chromosomes/plasmids have less than 11 genes). As seen in other studies the number of observed OGPs is correlated with the number of genes, with approximately one third of genes involved in overlaps, and for each species a large majority of co-oriented genes (603 in *E. coli*) (Figure 1). More interestingly, not all OGPs are independent from one another. In *E. coli,* for example, 1,186 unique genes form the 680 OGPs; so 174 genes are involved in more than one overlap (Figure 1). This property is observed across all bacteria considered here, and the number of OG triplets is correlated with the number of OGPs and so with the number of genes (Figure 1).

The observed number of genes involved in more than one overlap might simply be a byproduct of the creation of OG. To test this hypothesis we computed the expected number of genes involved in overlaps according to two sampling approaches. In a first case, we have sampled 680 adjacent gene pairs based on the *E. coli* genomic organization and computed the number of genes sampled more than once. In the second case, we have restricted the sampling to the co-oriented adjacent gene pairs based on the fact that most of the genes involved in OGPs are co-oriented. The expected number of genes involved in more than one OGP is 111 (sd = 8.7) and 92 (sd = 8), respectively, both smaller than the 174 observed in *E. coli* (t-test *p-*value *< 0.05*). Independent creation of OGPs thus cannot explain the observed abundances. This seems to support a possible biological function of OGP structures [4, 5, 14]. However, we argue that the number of genes involved in more than one overlap could be explained by taking into account genomic structures (see Discussion).

### Evolution of OGs

In comparison to the statistical characteristics of OGs, their evolutionary characteristics have received little attention: previous work suggests that genes involved in overlaps tend to be more conserved [4]. However, a large proportion of OGPs are part of operons (in *E. coli* 889 genes have their overlapping partner in the same operon). Because of this we have considered the evolution of overlaps in more detail. Using *E. coli* as a reference species we have identified orthologous genes in 698 bacterial species using reciprocal best hit and merged these data with information on genomic organization.

**Figure 2:**
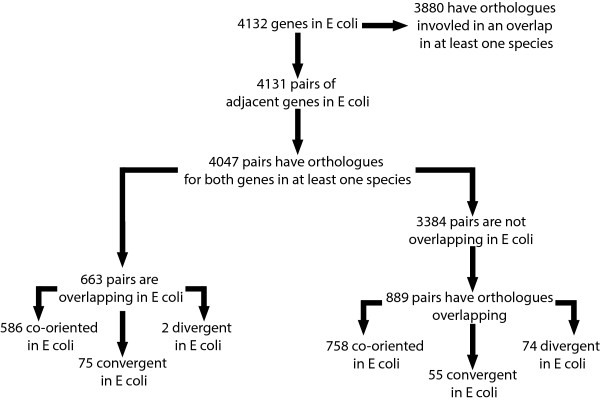
**Flowchart representing the categorization of the different genes pairs on** ***E. coli*** **in regard of different genomic organization and orthologues properties.**

The *E. coli* reference genome used here is composed of 4,132 protein coding genes, corresponding to 4,131 adjacent pairs of which 4,047 have at least one orthologue for each gene in the pair (out of the 4,132 protein coding genes annotated in *E. coli*, just 51 were orphans of orthologues.). Of these pairs 663 overlap (586 are co-oriented, 75 convergent and 2 divergent in *E. coli*). This leaves 3,384 adjacent gene pairs not overlapping in *E. coli*. From these non-overlapping pairs we can identify 889 adjacent gene pairs with orthologous genes overlapping in at least one other species. If we used the *E. coli* genomic organization as a reference these 889 pairs are grouped into the following categories: 758 co-oriented, 55 convergent, 74 divergent in *E. coli*. Interestingly, we lose the asymmetry between divergent and convergent overlapping gene pairs. This leaves 2,495 adjacent gene pairs in *E. coli* for which the orthologous genes are not overlapping in any other species of our phylogenetic panel. However, the fusion of the data corresponding to the overlapping genes identified in all the studied species on one hand, and the orthologous genes obtained with *E. coli* as a reference on the other shows that 3,880 of *E. coli* protein coding genes have orthologues involved in an OGP. In other words, non-adjacent genes in *E. coli* have orthologues forming OGP structures. To summarize, it is possible to characterize three groups of OGPs: genes overlapping in *E. coli* (approx. 1200 genes), genes adjacent in *E. coli* with orthologues involved in an overlap (approx. 1600 genes), and finally genes not adjacent in *E. coli* with orthologues overlapping in at least one species (approx. 1100 genes) (Figure 2). These observations hint that the evolutionary processes underlying genomic structures and OGPs in particular are complex.

**Figure 3:**
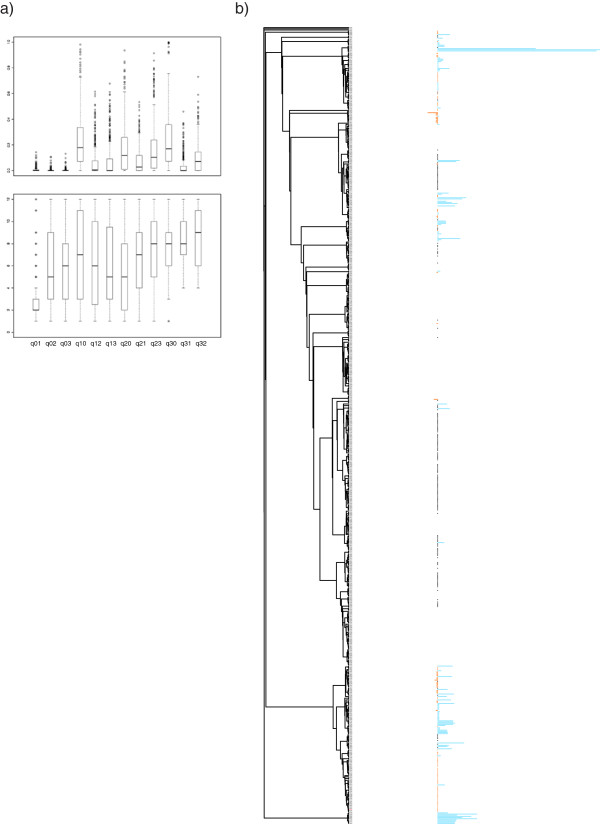
**Characteristics of overlapping genes evolution. a)** Representation of the transition rate obtained through the BayesTrait analysis of the orthologues of genes overlapping in *E. coli*. **Top** - Box plot representation of the normalized values extracted for each gene pairs. Because BayesTrait leads to the generation of transition rates with no upper limits, the normalization allow the comparison of transition rates for the different gene pairs. **Bottom** - Box plot representation of the order of the different rates (in ascending order) obtained from BayesTrait for each gene pair included in the analysis. **b)** Example of the observed configuration for one gene pair overlapping in *E. coli.* The results are presented on the phylogenetic tree build from the 23S rRNA sequences extracted from the different species of interest. The information related to the distance separating orthologues are coded as follow: The segment of DNA forming the overlap for overlapping orthologues is represente in orange, the portion of DNA separating adjacent orthologues is in blue, the orthologues pairs none adjacent are represented by a black dot, and if one or both of the orthologues is missing no mark in made. The length of the segments is proportional to the distance between the two genes of interest.

In order to get better insights into OG structure evolution, we study the evolution of 663 *E. coli* OGPs (Figure 3a) using models of overlap generation and loss (as well as gene gain and loss). The phylogenetic tree used to analyze the evolution of the different OGPs was produced using the 23S rRNA sequences extracted from each studied species. The largest observed transition rates correspond to the loss of one or both orthologous genes from any configuration. The rates at which overlaps are lost without the loss of the orthologues also tend to be high (more likely to be in the top 6 rates). The same analysis has been performed with a more restrictive definition of orthologs (50% of the largest protein need to be involve in the RBH). The results obtained with this new definition of orthologs (data not shown) show the same trends than the one presented here. Kolmogorov-smirnov tests comparing the distribution obtained for each rate with both type of orthologs reveal no significant differences. This suggests that the results are not sensitive to the definition of orthologs. Thus, overall, there is a high probability for overlaps to be lost. In addition, we analyzed the 663 trees with an extra layer of information (distance separating genes) using parsimony (Figure 3b). This confirmed the results obtained previously. In addition, we also observe variation of intergenic distance separating adjacent genes even between closely related species and, perhaps more surprisingly, of the length of overlap also between closely related species. These results show that these structures can be altered between closely related species (including variation of the length of the overlap itself) suggesting that OGPs are volatile structures. Based on these observations, it becomes apparent that if there is any biological advantage for two genes to overlap it can be loss/gained between closely related species, and that this lost/gain can be repeated independently for a given pair of genes. It also suggests that the mechanism leading to OG is relatively fast at the scale of evolution.

## Discussion

The different models put forward to explain the creation of overlapping gene structures are based on modification of the genes’ boundaries, with models in favour of either STOP [13] or START [14] codon positions changing as the main, or indeed sole, mechanism. We would like to start this discussion by reinforcing the idea that the movement of both START and STOP codons are needed to explain all observable OGP configurations; e.g. in order to create convergent and divergent OGPs changes of STOP, respectively, START codons are needed. The main question is associated with the mechanism leading to the creation of overlapping genes for co-oriented genes, especially since co-oriented overlapping genes represent the largest population of OGPs. Importantly, the different studies of OGPs published focused on the mechanism leading to their creation, but not to their loss. Our results suggest a relatively high turnover of the properties of adjacent gene pairs, including the creation and loss of overlap between coding sequence of proteins, and relatively rapid variation of the distance separating the coding sequences, including the size of overlaps. Because of these observations we believe that more work needs to be done in order to understand OGP evolution. Before progressing further with this discussion we would like to acknowledge that the analysis performed in this paper relies on the correct annotation of the different genes (just as any other bioinformatics study of OGPs, or other gene analysis in general). By using RefSeq, a non-redundant and curated database, we tried to minimize the risk of misannotated gene extremities/boundaries (and minimize the risk of misannotated overlaps along the way).

Because both a movement of START or STOP codons can lead to the creation of OGP in a co-oriented context, disentangling the mechanisms is challenging. However, if we assume that the different OGPs are created by the same mechanisms, the properties of the OGPs that are not co-oriented should give us relevant insights into the OGP formation mechanisms. Remarkably, there are more convergent than divergent OGPs (*p*-value ≈ 2.2e-16 < 0.05 obtained using Fisher’s Exact Test, which is not the case when all adjacent genes pairs are analyzed together), suggesting that the creations of different types of OGPs are not equivalent. The excess of convergent overlapping genes suggests that the movement of STOP codons is more frequent in the creation of OGPs. If the process leading to the creation of OGPs is independent of the orientation of the genes, this observation suggests that movement of STOP should be a predominant mechanism in the creation of OGPs. However, Cock et al. put forward the importance of the movement of START codons in the creation of OGPs for co-oriented genes OGPs based on the position of alternative START codons [14]. Therefore, if we assume that START codon movement is really a predominant mechanism leading to the creation of co-oriented OGPs, then negative selection probably acts against the movement of STOP codons. This aspect has, however, not been discussed previously. It is important to note that movement of STOP codons has been documented independently from overlapping genes [19]. This reinforces the idea that, in addition to being part of the evolution of coding genes, the movement of STOP codon is also likely to be part of the evolution of OGPs.

**Figure 4:**
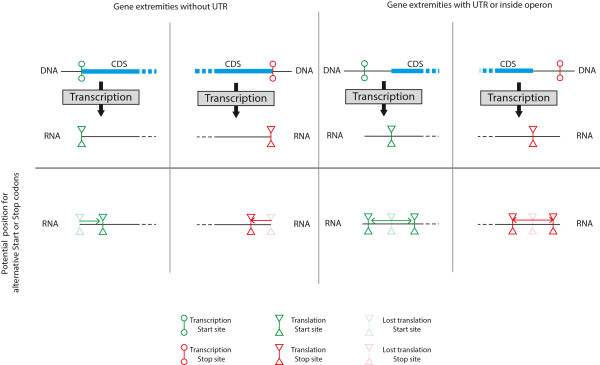
**Schema presenting the importance of UTRs in the freedom of movement of START and STOP codon position and by consequence the creation of OGP.**

Remarkably, published OGP studies looked only at the presence of alternative START and STOP codons on either side divorced from their genomic context. Protein coding genes are, however, not uniformly informative objects embedded within random sequences [20, 21]. Sequences surrounding amino-acid coding DNA also have biologically important functions (containing e.g. promoter regions, enhancers, hairpin structures); studying OGP creation divorced from their genomic context is bound to lead to misleading interpretations, and resolving this could potentially explain some of the contradictory results. Below we will discuss the possible importance of this additional information for the understanding of the high proportion of co-oriented OGPs. One important aspect to take into account is the link between transcription and translation. Proteins are produced in these two successive steps (transcription and translation) with initiation and termination steps that are highly regulated [22–25]. During the creation of OGPs, START or STOP codons are moved further away, respectively, into the 5’ or 3’ directions. In order for an alternative START or STOP codon to be used, it will have to be present in the transcribed mRNA. As shown in Figure 4, the presence of a UTR is required for the extension of the protein coding section of a gene. Without a UTR the loss of a START or STOP codon will lead to the use of an alternative START or STOP inside the transcribed regions. In this case, protein-coding genes without a UTR could not lead to the creation of OGPs. We know that at least some transcripts in bacteria have no UTRs [26]. A recent study in *Bacillus thuringiensis* has shown that out of the 1203 active transcript studied 16.3% were leaderless [27]. A more recent study on *E. coli* identified only 18 leaderless protein-coding genes out of 3,746 analyzed [28]. Even with a UTR, their length will lead to some restrictions regarding the possible position of usable alternative START or STOP codons. In *E. coli* and *K. pseudomonas* the vast majority of 5’UTRs are between 25 and 75 bp long [28]. The distribution of 3’ UTRs has also been studied in different bacteria, with lengths mainly between 25 to 50 bp, but with large variation between species [29]. Because of restrictions imposed by UTRs, the length of the overlaps should depend on the positions of alternative START and/or STOP codons and the length of the different UTRs associated to the genes.

In addition, intrinsic differences between START and STOP codons can lead to a bias in the creation of OGPs. The most obvious one is probably the importance of the START codon in the establishment of the reading frame of a gene. A change of position of a STOP codon should have little impact on the reading frame. Whereas changing the position of a START codon could lead to a reading frame change in two cases out of three. This could lead to a negative selection against OGP formation by START codon movement. Other signals (e.g. signals regulating initiation and termination of translation) could also impose further limitations. For example, an alternative START is unlikely to be used if it is upstream of the ribosome binding site. It is only once such additional information is taken into account that we will be able to fully grasp the properties of gene extremities movement.

This fact highlights that some properties, independent from the presence of alternative START and STOP codons, could lead to bias regarding the use of potential alternative START and STOP codons. The points made earlier are far from representing an exhaustive list of the possible sources of constrains regarding the creation and loss of OGPs. This just highlights the fact that studying OGPs by only looking at the properties of alternative START and STOP could lead to an incomplete understanding of their properties and evolution, which could potentially explain why we still lack of unifying model of OGP evolution.

The relative number of convergent and divergent OGPs gives us a potential insight into the mechanism leading to the creation of OGPs. We believe that the relative number of co-oriented OGPs compared to the other types of OGPs is also revealing. As stated before, in a system where all genes were under the same type of restriction on the creation of OGPs, the properties of co-oriented OGPs should be related to the properties of convergent and divergent OGPs. This is due to the fact that in co-oriented gene configurations, the first gene (according to the order of transcription) should behave like a gene in a convergent configuration, and the second like a gene found in a divergent configuration. In this case, the number of co-oriented OGPs should be equivalent to the number of convergent and divergent OGPs. However, co-oriented OGPs occur much more frequently than convergent and divergent OGPs (*p*-value ≈ 2.2e-16 < 0.05, obtained using Fisher’s Exact Test; this is also the case when all adjacent gene pairs are analyzed. However the ratio of co-oriented genes divided by the number of convergent plus divergent pairs is significantly larger for overlapping genes than for all adjacent pairs with *p*-value ≈ 2.2e-16 < 0.05, obtained using Kolmogorov-Smirnov test). No explanations have been proposed thus far for this large difference.

We have seen earlier how the relationship between transcription and translation can impact the creation of OGPs. The existence of operons could explain the large number of co-oriented OGPs [30]. In operons each gene has its own translation regulation signal but there is just one transcription initiation and one termination event. When a gene goes through an event leading to the creation of an overlap with one of the surrounding genes, the new coding sequence remains within the transcript and should not impact the regulation of transcription (with the exception of the movement of the START of the first gene or the STOP of the last). Therefore creation of OGPs inside operons could be more easily achieved than for genes transcribed into different mRNAs. This would explain the large number of co-oriented OGPs. In this operon configuration it is then possible to hypothesize that movement of the position of gene extremities could be relatively easily achieved, in the same way that the protein-coding sequence itself changes over time in all genes, leading to the observed plasticity of OGPs. Unfortunately, the lack of experimental characterization of operons in most species, in addition to the properties of the protocols used to predict operons (often relying solely on distances between coding sequences), make the assessment of this hypothesis challenging. A large-scale analysis of transcription profiles from different species will be needed to learn more about the possible impact of polycistronic RNAs on the creation of OGs.

Finally, the different points presented until now are associated to the creation of an overlap, which has been the main focus of the published work on OGPs. Our results show that OGP structures show high plasticity, stressing the need for a model describing the loss of OGPs. Here, a difference between the properties of START and STOP codon can provide a simple mechanism model based on the loss of OGPs. Moving the START codon downward the sequence, or the STOP codon upward, could lead to a loss of the overlap. However, the appearance of a START codon downward will not be enough. The best illustration of this is the use of ATG (the most frequent START codon) as a codon to encode methionine. For a START codon present inside the coding region of the gene to be used (a process that could lead to lead to a loss of overlap between OGPs) the original START needs to be lost. So for this process to lead to a loss of overlap or change in the length of the overlap two events are needed: the loss of the initial START codon and the acquisition of an alternative START inside the protein coding gene sequence in the relevant reading frame. Whereas, a STOP codon appearing upstream from an existing STOP codon one will be sufficient to lead to a shorter coding region independently of the loss of the original STOP. Once again, a change of STOP codon will have no impact on the reading frame. This suggests that movement of STOP codon is a likely process leading to the loss or change of length of overlap between adjacent genes.

At this stage, it is still difficult to appreciate fully all aspects involved in the evolution of OGPs. However, because our results suggests that OGPs are created and lost relatively easily it is only by building a model explaining gain and loss of OGPs that we will be able to understand overlapping gene structures.

## Conclusion

All genes probably see their START and STOP codons change over time (as a natural and probably common occurrence during evolutionary history) leading to frequent creation of alternative version of genes, in the same way that mutations lead to changes inside genes. This would represent a generalized version of the OGP creation model and is reinforced by a recent study showing shift of STOP codons even over short evolutionary distances [19]. In this model the presence of overlapping genes can then be seen as a by-product of sequence evolution and is not directly associated to a biological function as had been hypothesized (e.g. improving compression of the genome). This simple “neutral” model is in agreement with the high turnover of creation and loss of OGPs. This does not exclude, of course, the possibility that some of the OGs to have a biological function.

Our understanding of genome evolution is far from complete. Characterizing the different mechanisms associated to genome evolution, such as the creation of overlapping gene structures, will give us a better understanding of the important properties of biological systems and has the potential to improve fields such as synthetic biology; in this latter context in particular coordinating the expression of genes through such coupling is an attractive option to maintain control over the expression of different genes in response to simple transcriptional programs. More generally, such understanding is also essential to link genotypic and phenotypic evolution.

## Methods

### Sequences and annotations

The sequences and annotations for the 699 bacterial species (the genomic information is encapsulated in 759 chromosomes and 529 plasmids) considered here were retrieved from the “Genome Assembly/Annotation Projects” data from NCBI (ftp://ftp.ncbi.nih.gov/genomes/Bacteria/). For each species we used the refSeq annotation library. We decided not to apply any further filtering regarding genes or types of overlap in order to avoid the creation of bias in our dataset. Operon information was extracted from RegulonDB.

### Orthologue identification

A Reciprocal Best Hit (RBH) approach was used to identify orthologues (using Blastall 2.2.19, ftp://ftp.ncbi.nih.gov/blast/executables/LATEST/). Each protein of *Escherichia coli K12 MG 1655* was aligned against the full protein set of each of the 697 remaining bacterial species. The first hit of each Blast run was then aligned against the full set of *E. coli K12 MG1655* proteins. Two proteins were defined as potential orthologues if and only if the first hit of the second blast search corresponded to the protein used as a query in the first one [31].

To minimize the number of false positive these results were further filtered by demanding that the alignment had to involve more than 50% of the sequence of the smaller protein, and sequence identity had to be larger than 30%.

We have analyzed the difference in size for the genes identified as ortholog. Out of the 688570 ortholog pairs identified less than 1% have a substantial size difference (3× or more). In addition, 98% of the orthologs have a size difference smaller than 2 folds.

### Phylogenetic reconstruction

The phylogeny was inferred using the 23S rRNA [32]; one sequence was chosen randomly from each organism. This set of 23S rRNA sequences was aligned using MAFFT 6.611 (http://align.bmr.kyushu-u.ac.jp/mafft/software/) and the phylogeny was inferred using PhyML 2.4.4 (http://atgc.lirmm.fr/phyml/). Different alignments and phylogeny inference procedures resulted in very similar trees.

### Computation of the transition rates associated with overlapping pairs

For each OGP in *E. coli*, we characterized (and numerically encoded) the organization of the orthologues in each species as follows: overlapping (“3”), non-overlapping but adjacent (“2”), non-adjacent (“1”) and, finally, lacking orthologous gene for one or both genes (“0”). We then estimated the transition rates leading from one state to any other for all *E. coli* overlapping gene pairs using the BayesTrait software package (http://www.evolution.reading.ac.uk/BayesTraits.html), leading to a total of 663 matrices of the 12 transition rates. In order to compare results obtained from different pairs, each transition matrix was also normalized and we analyzed the inferred rates in order to infer which processes predominate the evolutionary dynamics of overlapping genes.

### Simulation of random gene pair sampling

We computed the expected distribution of OGPs under the assumption that the creation of overlapping gene pairs is random. This number is then compare to the observations in *E. coli*. Two particular sampling schemes were considered: (i) uniform random sampling over all *E. coli* gene pairs; and (ii) uniform random sampling over only the co-oriented genes pairs present in *E. coli*. In both cases 10,000 samples of 680 adjacent gene pairs were generated.

### Availability of supporting data

The tree supporting the results of this article is available in the treeBase repository: http://purl.org/phylo/treebase/phylows/study/TB2:S16248.
